# Dietary taurine effect on intestinal barrier function, colonic microbiota and metabolites in weanling piglets induced by LPS

**DOI:** 10.3389/fmicb.2023.1259133

**Published:** 2023-12-22

**Authors:** Dong-dong Zhao, Ye-dan Gai, Chen Li, Zi-zheng Fu, De-Qi Yin, Mingxin Xie, Jing-yuan Dai, Xin-xin Wang, Yan-xi Li, Gao-feng Wu, Ying Feng, Jian-min Hu, Shu-mei Lin, Jian-cheng Yang

**Affiliations:** ^1^Key Laboratory of Zoonosis of Liaoning Province, College of Animal Science and Veterinary Medicine, Shenyang Agricultural University, Shenyang, China; ^2^Animal Science and Technology College, Beijing University of Agriculture, Beijing, China; ^3^Animal Husbandry and Veterinary College, Jiangsu Vocational College of Agriculture and Forestry, Zhenjiang, China

**Keywords:** taurine, mechanical barrier, microbiota, metabolites, weaned piglet

## Abstract

Diarrhea in piglets is one of the most important diseases and a significant cause of death in piglets. Preliminary studies have confirmed that taurine reduces the rate and index of diarrhea in piglets induced by LPS. However, there is still a lack of relevant information on the specific target and mechanism of action of taurine. Therefore, we investigated the effects of taurine on the growth and barrier functions of the intestine, microbiota composition, and metabolite composition of piglets induced by LPS. Eighteen male weaned piglets were randomly divided into the CON group (basal diet + standard saline injection), LPS group (basal diet + LPS-intraperitoneal injection), and TAU + LPS group (basal diet + 0.3% taurine + LPS-intraperitoneal injection). The results show that taurine significantly increased the ADG and decreased the F/G (*p* < 0.05) compared with the group of CON. The group of TAU + LPS significantly improved colonic villous damage (*p* < 0.05). The expression of ZO-1, Occludin and Claudin-1 genes and proteins were markedly up-regulated (*p* < 0.05). Based on 16s rRNA sequencing analysis, the relative abundance of *Lactobacilluscae* and *Firmicutes* in the colon was significantly higher in the LPS + TAU group compared to the LPS group (*p* < 0.05). Four metabolites were significantly higher and one metabolite was significantly lower in the TAU + LPS group compared to the LPS group (*p* < 0.01). The above results show that LPS disrupts intestinal microorganisms and metabolites in weaned piglets and affects intestinal barrier function. Preventive addition of taurine enhances beneficial microbiota, modulates intestinal metabolites, and strengthens the intestinal mechanical barrier. Therefore, taurine can be used as a feed additive to prevent intestinal damage by regulating intestinal microorganisms and metabolites.

## Introduction

Diarrhea in piglets is an essential factor that hinders the healthy development of the swine industry. The causes of diarrhea in piglets are diverse (Yan et al., 2022). Numerous field studies have shown that the geographic location of the enclosure, the type of bacteria in the water source, weaning, material changes, and environmental stresses can affect piglet diarrhea (Quist et al., 2022). Immune stress caused by various environments and harmful pathogens is one of the most critical factors causing damage to the gastrointestinal barrier mucosa and leading to diarrhea in piglets (Bin et al., 2018). In many cases, antibiotics are used to treat piglet diarrhea, but problems such as antibiotic residues and drug resistance severely limit the beneficial development of the breeding industry (Ben et al., 2019). Moreover, due to the poor tolerance and drug resistance of piglets, the treatment effect of piglet diarrhea is not ideal (Wang et al., 2022). Therefore, it is more meaningful to take effective measures to prevent it than to treat it after the onset. To protect piglet’s health and produce environmentally friendly livestock products, researchers have been trying to find alternatives that can prevent diarrhea and avoid the use of antibiotics. Taurine has been tried to be used in animal production as an additive in animal feeds because of its advantages of safety, high efficiency, stable physical and chemical properties, and diverse functions (Wen et al., 2019). Taurine as a growth partner for piglets may be an effective countermeasure to prevent diarrhea in piglets since it can relieve stress for various reasons and protect intestinal mucosal cells.

Taurine is a compound widely present in livestock and poultry and plays an essential role in maintaining body osmolality, enzyme activity, receptor signal regulation, cell development, cell signaling, and cation homeostasis (Schaffer et al., 2010). Taurine is the second richest free amino acid among human colonic mucosa, accounting for about 18% of total free amino acids (Yang et al., 2017). Taurine is a good food attractant for aquatic animals, and when added to feed, it activates the animals’ appetite and significantly increases feed utilization (Brosnan and Brosnan, 2006). The mucosal integrity of the intestine, the largest immune and digestive organ in the animal body, is closely related to protecting the intestinal barrier function. The barrier function of the intestinal mucosa may be related to the fact that taurine increases the ratio of reduced to oxidized glutathione in the body. At the same time, from a histological perspective, taurine in moderate doses has been found to increase villi height in the jejunum and ileum of rats, increase the absorptive surface area in the gut, increase the weight of the intestinal mucosa in rats, and ultimately increase the metabolic rate of intestinal epithelial cells (Zhang et al., 2017).

Because piglets have underdeveloped intestinal tracts and weak digestive capacity, they are more susceptible to the effects of nutrients, feeding management, and environment during the weaning period, specifically, a sudden loss of appetite in weaned piglets, reduced resistance, a marked increase in diarrhea rates, and in severe cases, even death. Therefore, it is essential to prevent diarrhea in piglets at this stage to ensure the intestinal safety of weaned piglets. Under normal circumstances, the intestinal epithelium limits LPS entry into the intestinal barrier, protecting intestinal structure and function. The intestinal barrier refers to the barrier that can prevent pathogenic microorganisms, antigens, and bacteria from damaging the intestinal tract in the normal intestinal lumen, forming a layer of sequestration zones that can protect intestinal health and prevent detrimental substances from entering the blood circulation through the intestinal mucosa and transferring bacteria to cause a series of inflammatory responses in the body (Smith et al., 2010). Intestinal barriers can be divided into four types: mechanical, chemical, biological, and immune barriers (Clayburgh et al., 2004). In general, digestion and absorption of food in animals must be based on a stable intestinal environment and good permeability. The density of tight junction structures composed of intestinal mucosal epithelial cells directly indicates intestinal health. Occludin, Claudin-1, and ZO-1 consist of the tight junction proteins (Huber et al., 2001). The stability of tight junction structures depends primarily on the regulation of intracellular and extracellular signals and is influenced by food, microorganisms, and environmental stimuli. When tight junctions between epithelial cells of the intestinal mucosa are loosened, toxic substances are adsorbed on the intestinal mucosal surface, increasing the probability of intestinal disease infection (Gosain and Gamelli, 2005). If uncontrolled in time, it develops into a systemic inflammatory response syndrome (NagPal et al., 2006). However, under some stress conditions, the intestinal barrier function is severely impaired, cell permeability of LPS is reduced, and LPS is released in the adventitia, ultimately leading to acute inflammation of the organism through resident immune cells (Abreu, 2010). Inflammatory bowel disease (IBD) is an immune-mediated chronic gastrointestinal disorder classified as Crohn’s disease (CD) and ulcerative colitis (UC). It is mainly caused by an immune reaction between food antigens in the intestine and the intestinal microbiota, resulting in impaired mucosal immune function and disruption of the intestinal symbiotic ecosystem (Moriello et al., 2022). Numerous factors influence susceptibility to enterocolitis, and these factors are often reported to interact with one another. For example, congenital and adaptive immune system abnormalities, intestinal microbial imbalances, genetic factors, and environmental factors are considered as the leading causes of intestinal inflammation (Tamburini et al., 2022).

The intestine possesses the largest surface among the human organs in the whole body. Microbial cell densities in the intestine have been reported to be approximately 1 trillion per milliliter of intestinal fluid. At the same time, the number of microbiota present in each part of the digestive tract varies widely. For example, the type and number of bacteria in the large intestine are typically greater than those in the stomach and small intestine (Jia and Jeon, 2019). The numerous microbiota play roles in host nutrition, immunity, metabolism, anti-infection, and intestinal stability. In addition, dysfunction of the microbiota is thought to be associated with adverse effects such as dysfunction of intestinal mucosal cells, impaired intestinal immune system, and uncontrolled intestinal inflammation (Ramezani and Raj, 2014). Intestinal microbiota has proven to have far-reaching significance in maintaining host health and explaining disease development. The benefits of probiotics have been gradually recognized since people began eating yogurt, cheese, and fermented milk (Pavlović et al., 2012). Probiotics are a type of live microorganism that can benefit the host when the body consumes enough of them (Canfora et al., 2015). since the 20th century, Metchnikoff has been working with *Streptococcus thermophilus* and *Lactobacillus delbrueckii*, and others that benefit the body by affecting the bacterial microbiota in the digestive tract (Plaza-Diaz et al., 2019). Probiotics may also exhibit antagonistic effects against pathogenic microorganisms by stimulating the immune system (Gómez-Llorente and Muñoz, 2010). Taurine is a type of natural antimicrobial substance and plays a critical role in intestinal immune processes as an essential defense system for microbes (Sannasiddappa et al., 2017). Taurine is used to form conjugated bile acids, which are later degraded by intestinal microorganisms. These bile acids play a crucial role in controlling the gut metabolism, molecular signaling, and influencing microbial composition in biological processes (Guzior and Quinn, 2021). Weaning stress can easily cause diarrhea, reduced feed intake and conversion, and poor piglet growth performance. Therefore, sub-therapeutic antibiotics such as chlortetracycline are widely used in the breeding industry to relieve piglet weaning pressure and promote growth. By contrast, the global use of antibiotics implies serious problems, including the increase of resistant bacteria and antibiotic residues, which can cause serious harm to humans and other biological health. As a result, many countries have begun to restrict or even ban antibiotics as feed additives to promote growth. Therefore, it is essential to develop feed additives that have no side effects as alternatives. Our research team has found that adding 0.3% taurine in the diet can significantly reduce the diarrhea rate of piglets induced by LPS, but the specific mechanism is unknown. To explore this mechanism, we conducted this study to determine if taurine could play a role by altering the intestinal barrier and intestinal microbes and metabolites.

## Materials and methods

### Animals and experimental design

Eighteen weaned piglets (28 days old, 9.05 ± 0.5 kg) were selected as test subjects. They were randomly classified into 3 groups, with 6 replications per group. Piglets were maintained as pairs in metal floor cages at 50%–65% relative humidity and 20°C–25°C. They were the control group (CON group, basic diet + intraperitoneal injection of equal volume of saline), the treatment group (LPS group, basic diet + intraperitoneal injection of 100 μg/kg-BW of LPS), and the prevention group (TAU + LPS group, basic diet + 0.3% taurine + intraperitoneal injection of LPS). According to the previous experiment, the feeding cycle for this experiment is 28 days (Ma et al., 2022). LPS was injected after 28 days of feeding. Feeding will occur daily at 9:00 and 16:00. The standard of the essential diet follows the feeding standard of the NRC (2021). After the study, composition of basic feed and nutrient levels is shown in Table 1.

**Table 1:** Composition and nutrient levels of the basal diet.

Items	Content (%)
**Ingredients** ^a^	
Corn	30.00
Puffed corn	20.00
Peeling soybean meal	14.00
Expanded soybean meal	5.00
Fermented soybean meal	5.00
Steam fish meal	3.50
Sucrose	5.00
Whey powder	10.00
Limestone	1.00
CaHPO_4_	1.10
NaCl	0.30
Soybean oil	2.00
Lysine hydrochloride	0.35
Met	0.05
Thr	0.18
Trp	0.02
Choline chloride (50%)	1.00
Compound acidifier	0.50
Premix^b^	1.00
Total	100.00
**Nutrient levels** ^c^	
CP	20.00
Ca	0.90
AP	0.45
Lys	1.35
Met	0.38
Sulfur-containing amino acids	0.76
Thr	0.86
Trp	0.22
ME/(MJ/kg)	14.50

a The ingredient content was air-dried basis.

b The premix provides per kilogram of feed: VA 12000 IU, VD3 3000 IU, VE 60 mg, K33 mg, B2 7.5 mg, B6 4.8 mg, B12 0.04 mg, niacinamide 45 mg, pantothenic 30 mg, folic acid 1.2 mg, biotin 0.22 mg, Fe 120 mg, Cu 15 mg, Zn 120 mg, Mn 50 mg, Se 0.3 mg, I 0.6 mg.

c The chemical composition was measured, while metabolic energy was calculation based on the Chinese Feed Composition and Nutritional Value Table (29th edition, 2018). The nutrient compositions was dry matter basis.

### Growth performance and apparent digestibility

On the 1st and 28th days of the trial period, the fasting weight of the weaned piglets was recorded morning at 6:00, and the daily gain of each piglet was calculated ADG. After feeding, the piglets’ remaining daily feed was weighed, the daily feed intake (ADFI) was calculated and feed to weight ratio (F/G) was calculated.

In the last 3 days of the experiment fecal samples were collected daily from each replicate and merged in each replicate. Approximately 600 g samples were taken, dried at 65°C for 96°C hours, and ground into fine powder for apparent total digestibility analysis. According to the AOAC Association’s method (citation 2000), the composition of dietary and fecal samples was measured, including dry matter (DM, method 930.15), crude protein (CP, method 976.06), and ether extract (EE, method 920.39). Acid insoluble ash (AIA) was used as an internal marker and determined according to the method described by Xie et al. (2021).

### Sampling

After bleeding, serum was collected from piglets. On day 28 of the experiment, after 12 h of fasting, LPS-injected piglets (3 h later) were carefully transferred from their cages to an adjacent slaughterhouse, immediately euthanized by administration of sodium pentobarbital solution (4%, 40 mg/kg) into the jugular vein (Ma et al., 2022). All animal samples were quickly collected within 2 h. The entire digestive tract was dissected into the stomach, duodenum, jejunum, ileum, cecum, and colon according to anatomic lines. 2 centimeter intestinal sections were created from the colon tissues and placed in 4% paraformaldehyde fixative for histological observation by electron microscopy. The remaining colon samples and fecal contents were placed in sterilized tubes and stored at −80°C freezer.

### ELISA assay

Serum diamine oxidase (DAO) was determined using an enzyme-linked immunosorbent assay kit (Jiangsu Meiman, China) and the test was analysed according to the manufacturer’s recommended instructions.

### Analysis of intestinal morphology

The hematoxylin-eosin (HE) staining and scanning electron microscopy procedures were performed based on previous studies (Zhao et al., 2023). Scanning electron microscopy procedure: (1) cut out a completely fixed tissue, place it in an embedding box, and wash the fixed solution with clean water (30 min). (2) Wash thoroughly with distilled water for 1 h (at 4°C), and rinse thoroughly with PBS 3 times (5 min each time). (3) Wash the samples thoroughly with tannin acid water (2%) for 3 h at 4°C, and rinse thoroughly with PBS 3 times (5 min each time). (4) Place in fixative solution (30 min at 4°C) and rinse thoroughly with double distilled water. (5) Dehydrate in alcohol from low to high concentration for 15 min each time. (6) Place the colon sample in isoamyl acetate twice for 15 min. (7) Place the sample in a dryer to dry. (8) Fix the sample with conductive adhesive and coat it with a metal coating device. (9) Finally, the coated sample was photographed with a scanning electron microscope.

### Preparation of RNA and RT-qPCR

RNA purification was performed according to the manufacturer’s procedure via RNAiso plus (Takara, Dalian, China), and PrimeScrip II First Strand gene synthesis kit (Vazyme, Nanjing, China), RT-qPCR was operated by the StepOne Plus real-time polymerase chain reaction system (Applied Biosystem, Carlsbad, United States) followed by HiScrip II one-step RT-qPCR SYBR Green Kit (Vazyme, Nanjing, China) according to the manufacturer’s procedures. The NCBI Primer-BLAST tool was used for designing oligo DNA primers. Supplementary Table S1 shows the sequences for the primers of target genes. β-actin, a housekeeping gene, was used for normalization, and their abundance was estimated by the 2^−ΔΔCt^ method. Triplicated samples were analyzed for all experiments.

### Immunohistochemical analysis

The harvested colon was fixed in formalin (4%), and paraffin was used for embedding samples. Serial sections were used for immunohistochemical analysis every 25 sections. For antigen retreat, Tissue sections were boiled in sodium citrate (10 mmol/L, pH 6.0) for 10 min at 100°C and sections were blocked with BSA (Sigma, St. Louis Missouri, United States). The sections were then incubated with primary antibody solutions for ZO-1 (Affinity, Jiangsu, China), Claudin-1 (Bioss, Beijing, China), and Occludin (Affinity, Jiangsu, China). Anti-rabbit antibodies conjugated with fluorescence dyes (Alexa 594 and Alexa 488) were used as the secondary antibody (Boster, Wuhan, China).

### DNA preparation and sequencing of 16s rRNA gene

Porcine colonic contents were used for the extraction of genomic DNA from microbes under sterile conditions by the E.Z.N.A ^®^ Stool DNA Kit (Omega Inc., Norcross Georgia, United States). The extracted DNA quality was detected by electrophoresis with agarose gel and spectrophotometry. PCR amplification was performed by 515F primer (5′-GTGYCAGCGCGTAA-3′) and 806R primer (5′-GGGACTACNVGGTWTCTAA-3′) with a slight modification of the small subunit of thermophiles (V4 region of 16s of bacteria and animal). The specific barcodes for the general primer were used for relabeling the 5′ ends of the primers for sampling and sequencing. The Illumina Miseq platform was utilized for DNA sequencing following the manufacturer’s protocols from LC-Bio. The raw data were first screened and sequences were removed from consideration if they were shorter than 230 bp, had a low quality score (≤20), contained ambiguous bases or did not exactly match to primer sequences and barcode tags, and separated using the sample-specific barcode sequences. Qualified reads were clustereds into operational taxonomic units (OTUs) at a similarity level of 97% (Edgar, 2013) use Uparse algorithm of Vsearch (v2.7.1) software. The Ribosomal Database Project (RDP) Classifier tool was used to classify all sequences into different taxonomic groups against SILVA128 database (Cole et al., 2009). Alpha diversity (Chao1, Shannon) was calculated using QIIME software version 1.8.0 and analysed by PCoA for clustering, which reflects bacterial diversity and presence. In this experiment, species distributions at the phylogenetic, class, and family levels were analyzed using column charts. To highlight significant species, LEfSe Effect Size analysis can be used to achieve a comparison of multiple groups. Firstly, Kruskal–Wallis was used to detect the species with significant differences in abundance between different groups, and the threshold was set to 0.05. Then, Wilcoxon rank sum test was used to test the consistency of the differences in the sub-groups of different groups. Finally, linear regression analysis (LDA) was used to estimate the effect of each species abundance on the difference effect and set the threshold to 3 (Segata et al., 2011).

### Metabolomics analysis

In this experiment, three fecal content samples were collected, and six independent replicates were used in each group. First, 50 mg of content sample from each sample is weighed and placed in a 2 mL EP tube containing 200 μL DEPC water and 480 μL methyl tert-butyl ether. Then, put a steel ball in the EP tube, ground it with a grinder (45 Hz) for 4 min, and repeat this process thrice. The above sample was kept in a freezer at −20°C for 1 h, and 300 μL of the supernatant solution was placed in an EP tube and aspirated. Then the sample was dried in a vacuum concentrator and added methyl tert-butyl ether for redissolution after drying. UHPLC by using Agilent 1,290 Infinity II series UHPLC system was performed with a non-fixed column of 100 × 2.1 mm and 1.7 μm. DeVice (Agilent Technologies) has a non-fixed column of 100 × 2.1 mm and 1.7 μm. The mobile phase consisted of ammonium formate (10 mmol/L) and ammonia (10 mmol/L), followed by the mobile phase was acetonitrile. The column temperature was estimated to be 35°C. The temperature of the autosampler was set to 4°C, and the injection volume was set to 1 μL.

### Statistical analysis

Experimental results are reported as means ± SD over at least three replications. SPSS (v22) statistical software tool (IBM, Armonk, NY, United States) was used to check and evaluate the results’ significance. GraphPad Prism (v7.0) was used to display the data graphically. ANOVA for single factor analysis of variance was utilized to identify the differences in the data between the two groups were offset as *p* < 0.05 (significant differences), *p* < 0.01 (highly significant differences), and *p* < 0.001 (super highly significant differences).

## Results

### Growth performance and tissue observation

**Figure 1:**
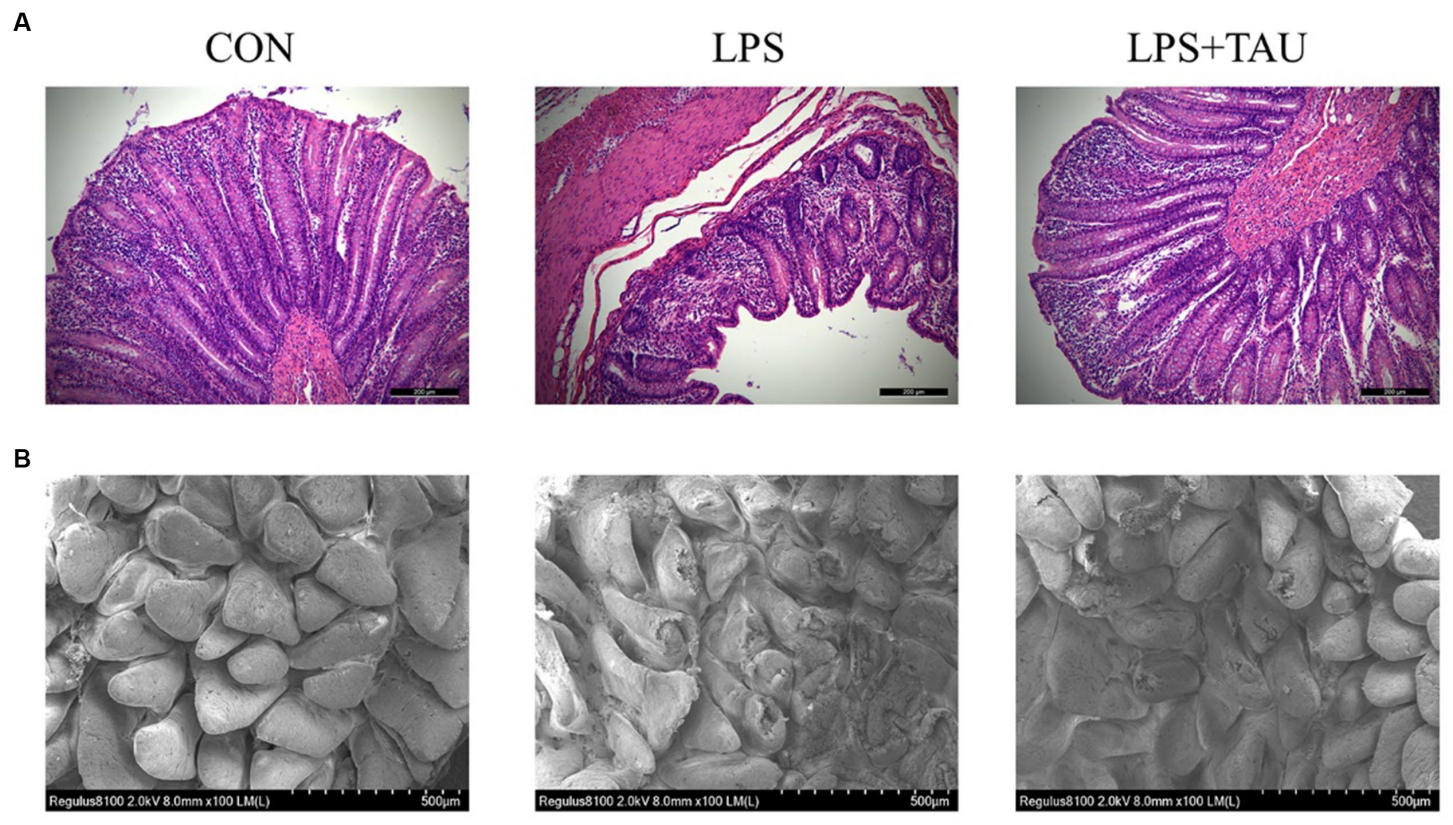
Taurine effect on weaned piglets’ colon morphology. **(A)** Representative image of colon sections with hematoxylin and eosin staining. The scale bar denotes 200 μm. **(B)** Representative image of colon sections by transmission electron microscopy. The scale bar denotes 500 μm. All data are shown as mean ± SD (^*^p < 0.05 and ^**^p < 0.01). (*n* = 6 samples/group).

All piglets remained healthy during the experimental period without diarrhea before the LPS injection. There was no difference in ADF1 during the experimental period. However, ADG in the group for taurine-fed was markedly higher compared with ADG in the group not given taurine (*p* < 0.05). The F/G results showed that the TAU group was significantly lower than the CON group (Table 2). The apparent digestibility of feed ASH and P increases linearly with the increase of taurine supplementation level (*p* < 0.05). There was no significant difference in the apparent digestibility of other CP, EE, and Ca (Table 3). Intestinal morphology showed sparsely arranged colonic villi and shorter length in the LPS group compared with the CON group; the group of TAU + LPS showed significant improvement (Figure 1A); the intestinal villi in the CON group were well developed, flat and round with clearly visible microvilli, while the LPS group showed many folds and indentations on the villi surface. Intestinal villi in the TAU + LPS group were markedly improved compared with those in the LPS group (Figure 1B).

**Table 2:** Effects of dietary taurine on growth performance of weaned piglets.

Group	Initial BW (kg)	Finial BW (kg)	ADG (g)	ADFI (g)	F/G (g/g)
CON	8.90 ± 0.13	23.62 ± 0.63	525.3 ± 18.9^a^	691 ± 3.5	1.34 ± 0.05^a^
TAU	9.05 ± 0.09	24.42 ± 0.25	548.7 ± 0.74^b^	707 ± 0.02	1.28 ± 0.02^b^

^a,b^Values in the same row with different superscripts are significantly different (*p* < 0.05).

**Table 3:** Effects of dietary taurine on apparent digestibility of weaned piglets.

Item	CP	EE	ASH	Ca	P
CON	79.49 ± 0.17	71.26 ± 0.25	57.03 ± 0.44^a^	59.39 ± 0.28	38.92 ± 0.54^a^
TAU	80.46 ± 0.62	72.19 ± 0.56	60.04 ± 0.10^b^	60.00 ± 0.79	40.29 ± 0.76^b^

^a,b^Values in the same row with different superscripts are significantly different (*p* < 0.05).

### Intestinal physical barrier function

**Figure 2:**
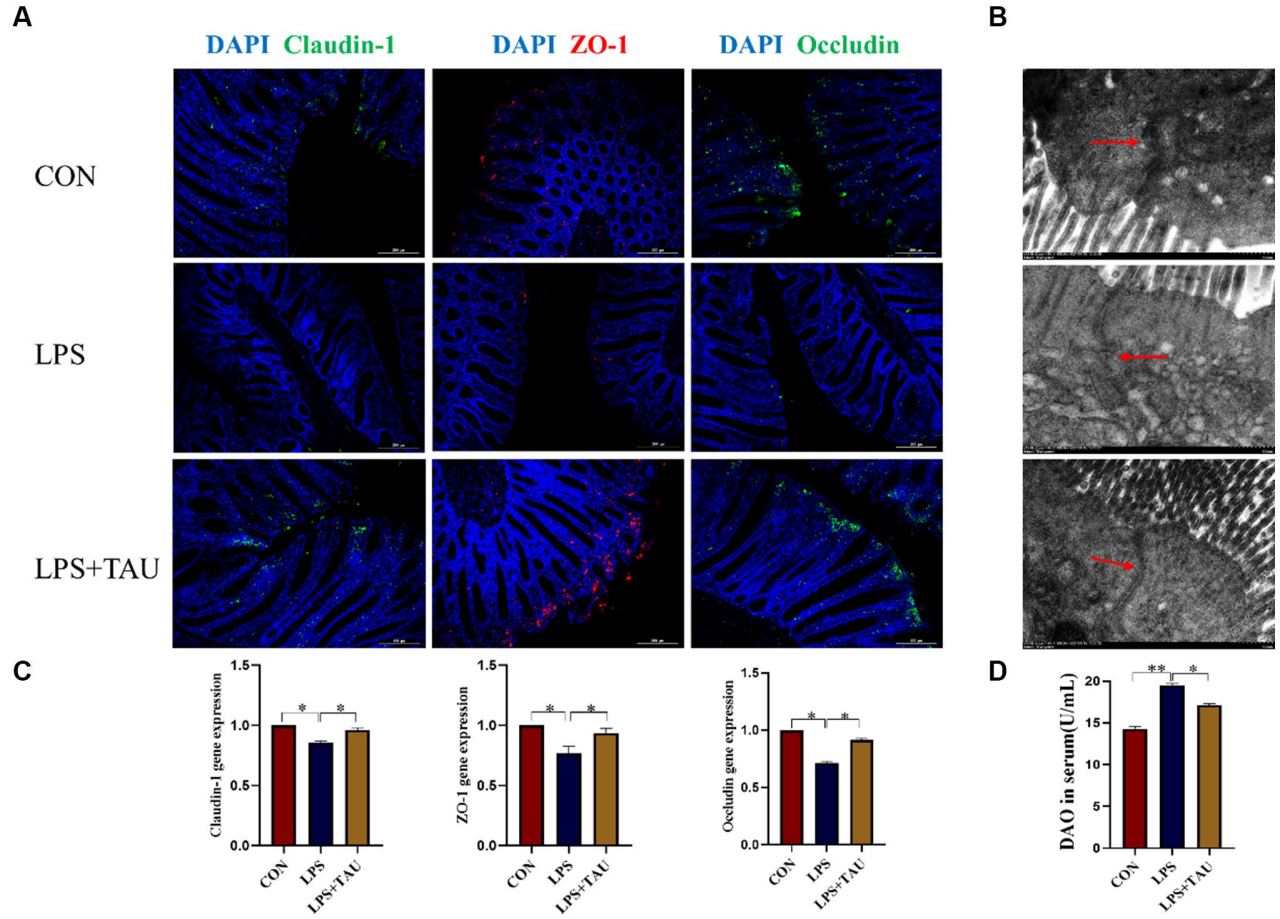
Effect of taurine on intestinal mechanical barrier function. **(A)** Relative expression of Claudin-1, ZO-1 and Occludin protein analyzed by immunohistochemical fluorescence. The scale bar denotes 200 μm. **(B)** Transmission electron micrograph of colon tight connections of weanling piglets. The scale bar denotes 500 μm. **(C)** Relative expression of *Claudin-1*, *ZO-1* and *Occludin* mRNA was analyzed by RT-qPCR. **(D)** DAO in serum of weaned piglets. Data are means ± SD. ^*^p < 0.05. (*n* = 6 samples/group).

The results showed that the expression levels of Claudin-1, ZO-1 and Occludin for tight junction proteins in the colon of the TAU + LPS group were markedly up-regulated compared to the LPS group (Figure 2A). To validate the above results, transmission electron microscopy revealed more extended adhesion junctions and deeper desmosomes in the TAU + LPS group compared with the LPS group (Figure 2B). In addition, expression of *Claudin-1*, and *ZO-1*, and *Occludin* mRNA in the colon of the TAU + LPS group were markedly up-regulated compared with the LPS group (Figure 2C). The DAO content in serum was markedly up-regulated in the group of LPS compared with the group of CON (*p* < 0.01) and markedly down-regulated in the group of TAU + LPS compared with the group of LPS (*p* < 0.05) (Figure 2D)

### Colon microbiota

**Figure 3:**
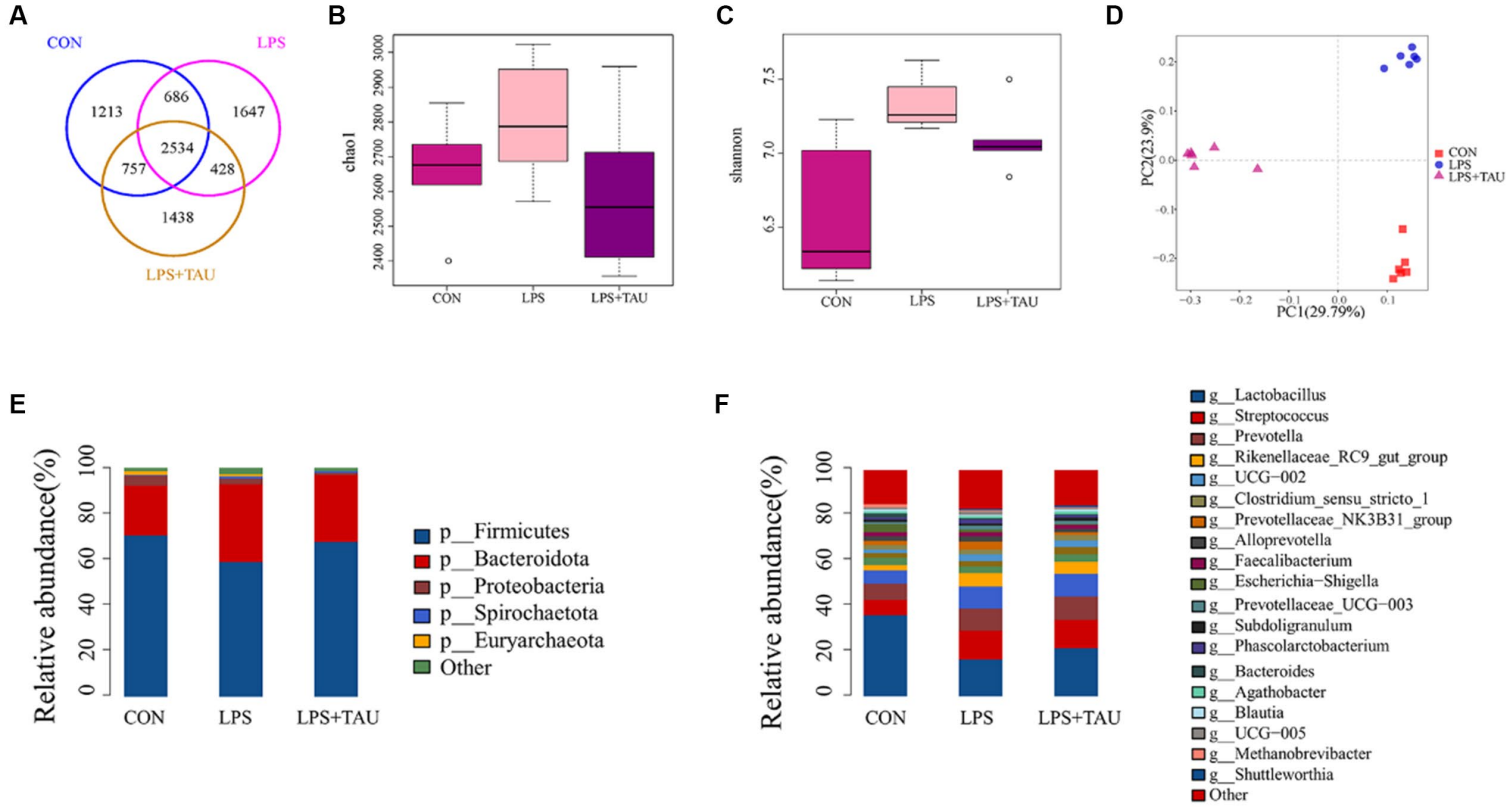
Statistical analysis of sequencing results and diversity. **(A)** Venn diagram demonstrates that OTUs are shared among the colon. **(B,C)** Shannon and Chao1 indexes for the 6 segments of the intestine. QIIME1 test to analyze the significance of differences for each index, ^*^p < 0.05 and ^**^p < 0.01. **(D)** Principal coordinate analysis (PCoA) based on the total sample. **(E)** Microbial composition at the level of intestinal segments. **(F)** Microbial composition of intestinal segments. (*n* = 6 samples/group).

Among the four sample groups, OTUs were clustered when sequence similarity was more than 97%. The number of OTUs in each group is shown in the Venn diagram, and a total of 8,703 OTUs were counted. There were 1,213 unique OTUs in the CON group, 1,647 unique OTUs in the LPS group, 1,438 unique OTUs in the TAU + LPS group, and a total of 2,534 shared OTUs in all groups (Figure 3A). Intestinal bacterial diversity was further analyzed using the Chao1 and Shannon indexes. The outcomes demonstrated a marked up-regulation in microbial abundance in the LPS group compared with the CON group (*p* < 0.05) and a marked down-regulation in species richness and diversity in the TAU + LPS group compared with the LPS group (*p* < 0.01) (Figure 3B). Compared with the CON group, the microbial concentration in the LPS group was markedly up-regulated (*p* < 0.01) and the species concentration and diversity in the TAU + LPS group were markedly down-regulated (*p* < 0.05) (Figure 3C). The PCoA analysis chart showed that the contribution of the two principal components was 23.9% and 29.79% respectively, and indicated that the microbial composition and structure of the three groups were very different and relatively obvious (Figure 3D). The relative abundance of the different phyla and genera is shown in Figures 3E,F. At the phylum level, the relative amount of *Firmicutes* in the LPS group (58.67%) was markedly down-regulated compared with the CON group (70.45%) (*p* < 0.01), and that of the TAU + LPS group (67.45%) was markedly increased compared with the LPS group (*p* < 0.01). For *Spirochaetota* and *Bacteroidota*, the LPS group (*Spirochaetota* 1.05%, *Bacteroidota* 34%) had markedly increased abundance compared to the CON group (*Spirochaetota* 0.6%, *Bacteroidota* 21.65%) (*p* < 0.01). In contrast, the relative abundance of TAU + LPS group (*Spirochaetota* 0.74%, *Bacteroidota* 29.52%) had significantly lower abundance compared to the LPS group (*p* < 0.01) (Figure 3E). At the genus level, the relative amount of *Lactobacillus* was markedly up-regulated in the TAU + LPS group (21.19%) compared with the LPS group (16.19%) (*p* < 0.05) (Figure 3F). The results for each sample size are shown in Supplementary Figure S1.

**Figure 4:**
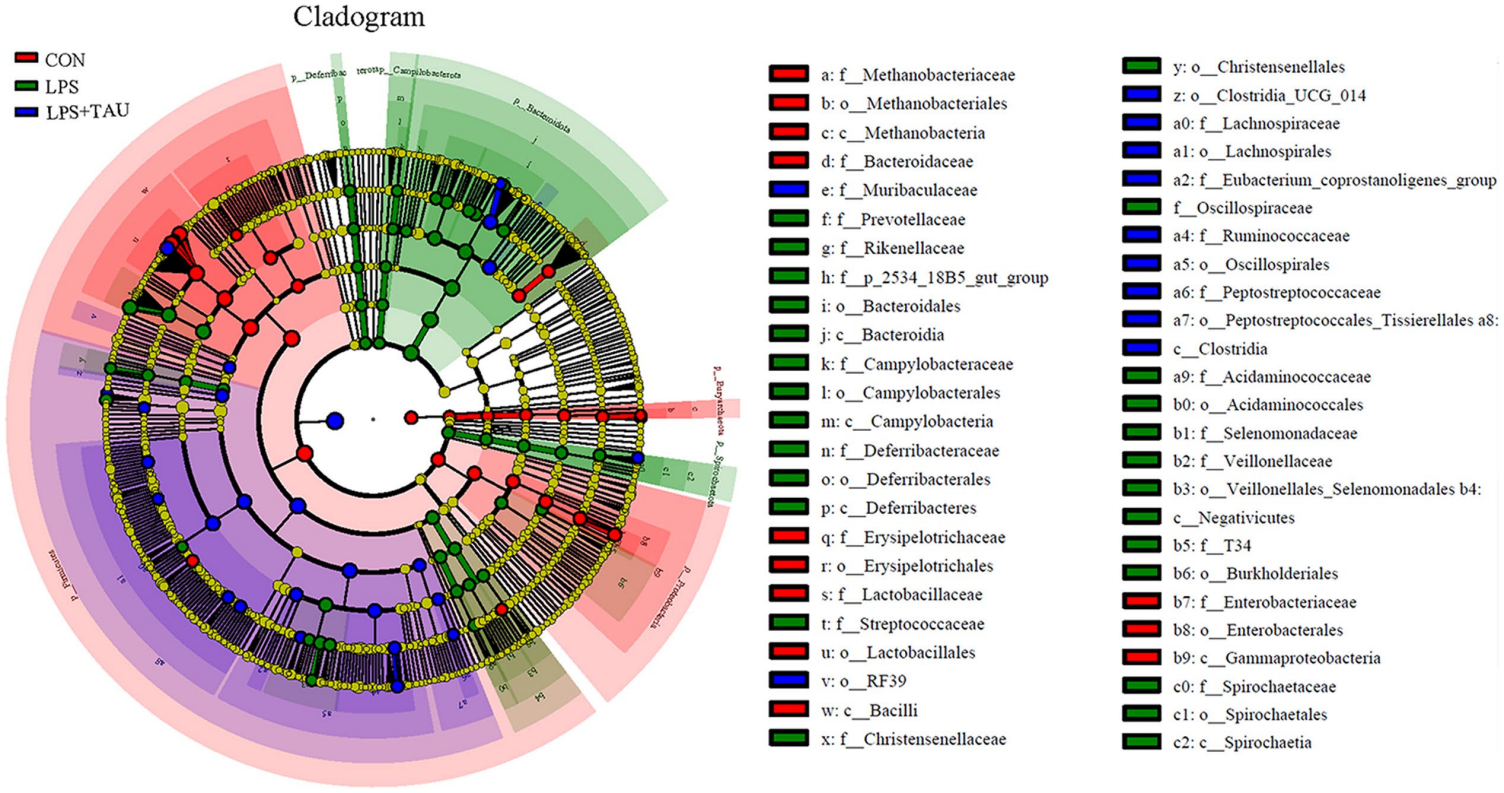
Differences in microbial abundance between different groups. Cladogram of LEfSe analysis (*n* = 6 samples/group).

By using the LDA Effect Size analysis method, LDA scores of 3 or higher in the bacterial taxa were selected as biomarker taxa, and as shown in the figure, the probiotic *Lactobacillus_reuteri* (LDA = 3.81, *p* = 0.00156) was significantly up-regulated in the TAU + LPS group and occupied a major position among the altered species in this group. At the same time, the predominant bacteria in the TAU + LPS group were *Clostridium_sensu_stricto_6* (LDA = 3.29, *p* = 0.00076), *Eubacterium_coprostanoligenes_group* (LDA = 3.77, *p* = 0.00186), *Ruminococcaceae* (LDA = 3.81, *p* = 0.00156) and *wallaby_gut_metagenome* (LDA = 3.10, *p* = 0.00309) (Figure 4). The percentage of the various groups that were identified as markers are shown in Supplementary Table S2.

### Colon metabolome

**Figure 5:**
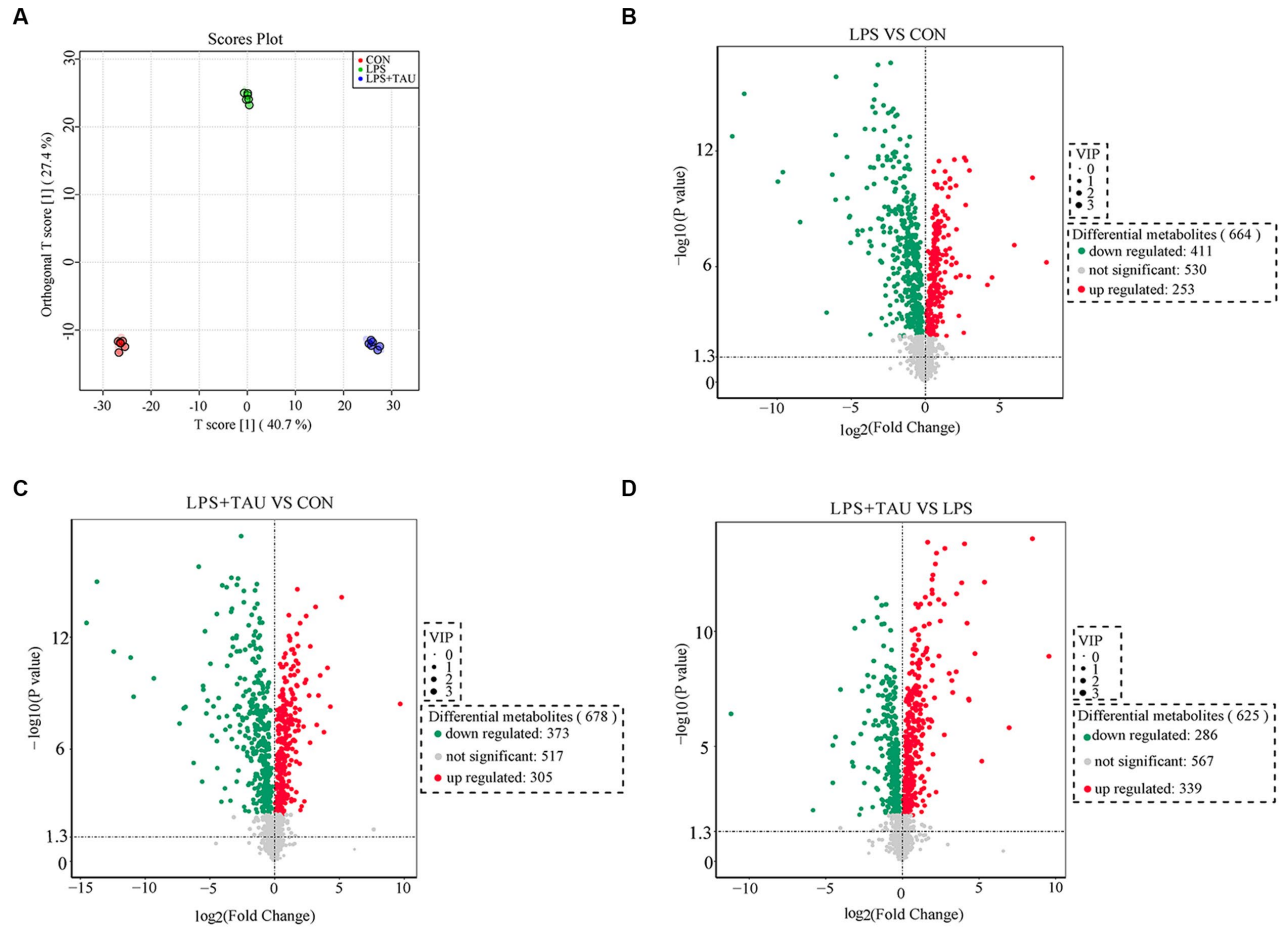
Changes in colon metabolome. **(A)** Modelling the relationship between metabolite expression and sample grouping using partial least squares regression. **(B–D)** Changes in porcine colon metabolites of CON group, LPS group and TAU + LPS group. (*n* = 6 samples/group).

To further understand the changes in colonic metabolism in piglets after LPS and TAU treatment, a non-targeted metabolomic analysis of colonic contents was performed. Partial least squares discriminant analysis yielded accurate results. Responses were relatively well differentiated between groups, with little within-group variability (Figure 5A). The criteria for screening for differential metabolites in this test are *p*-value <0.05 and VIP ≥1. A volcano plot was used to visualize the differential metabolites from the screening outcomes. The vertical axis indicates the student’s *t*-test *p*-value (logarithm with 10 as the bottom) in Figure 5. In the LPS group, 411 metabolites were down-regulated, and 253 metabolites were up-regulated compared with the CON group (Figure 5B); in the TAU + LPS group, 373 metabolites were down-regulated, and 305 metabolites were up-regulated compared with the CON group (Figure 5C); in the TAU + LPS group, 286 metabolites were down-regulated and 339 metabolites were up-regulated compared with the LPS group (Figure 5D). Data on the significantly different metabolites of interest to us in the LPS and LPS + TAU groups are shown in Supplementary Table S3.

**Figure 6:**
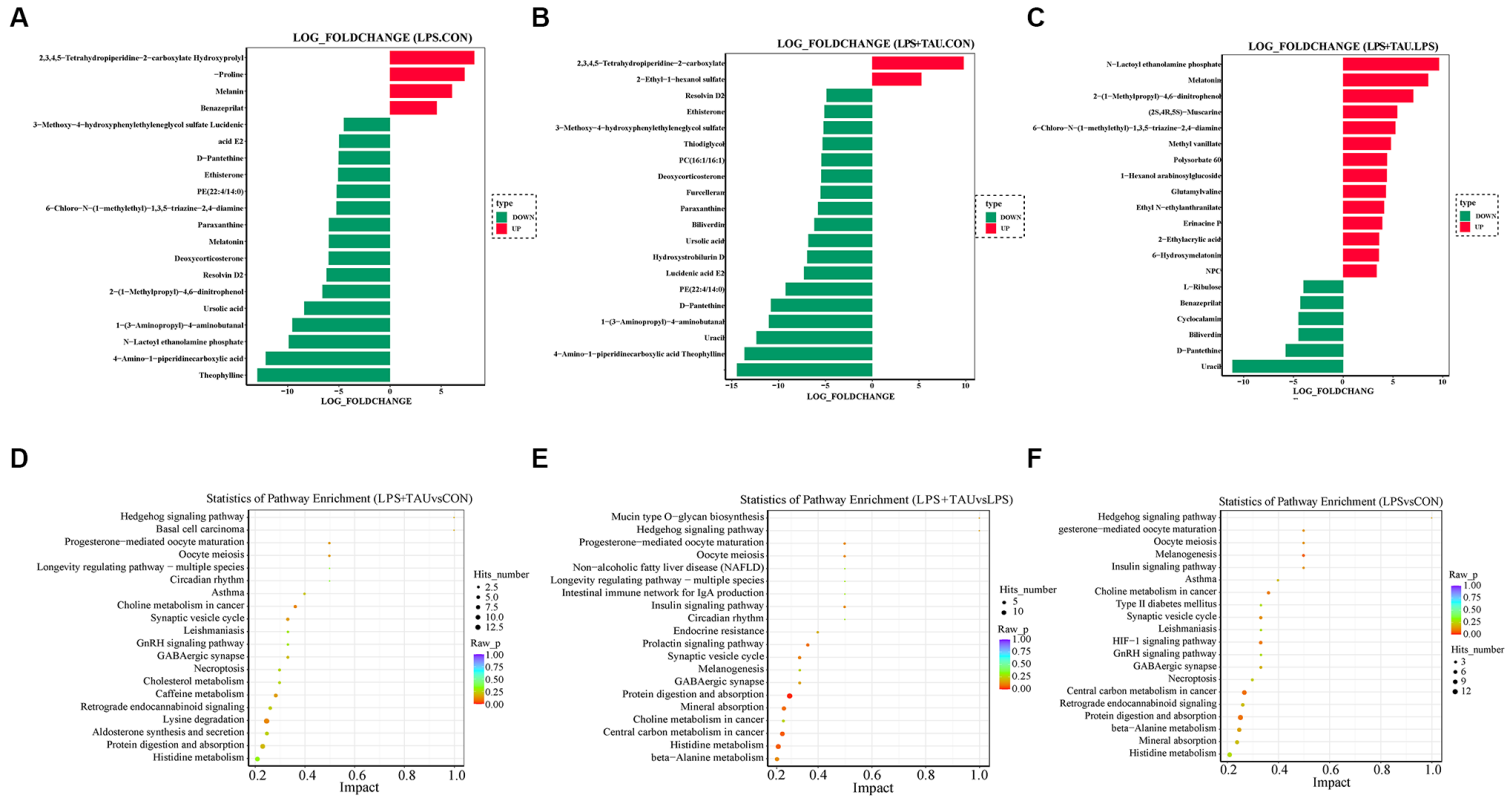
Comparison of colonic metabolites in each group of pigs. **(A–C)** Top 20 differential metabolites between pig groups (*n* = 6 samples/group). **(D–F)** Metabolic pathway analysis for each comparator combination (*n* = 6 samples/group).

The top 20 metabolites that differed through the collection of fecal contents from 18 samples are shown in the Figure 6. The figure shows that *Melatonin*, *6-Chloro-N-(1-methyl ethyl)-1,3,5-triazine-2,4-diamine*, 2*-(1-methyl propyl)-4* and *6-dinitrophenol* were markedly down-regulated and *Benazeprilat* was markedly up-regulated in the LPS group compared with the CON group (*p* < 0.01). In the TAU + LPS group, the content of the above 5 metabolites was markedly altered compared with the LPS group (*p* < 0.01) (Figures 6A–C). Compared to the CON group, the specific metabolic pathway of LPS group pigs is protein digestion and absorption, while the specific metabolic pathway of TAU + LPS group pigs is lysine degradation; compared with the LPS group, TAU + LPS group pigs are primarily involved in protein digestion and absorption and histidine metabolism (Figures 6D–F).

### Correlation analysis of gut bacteria and metabolites

**Figure 7:**
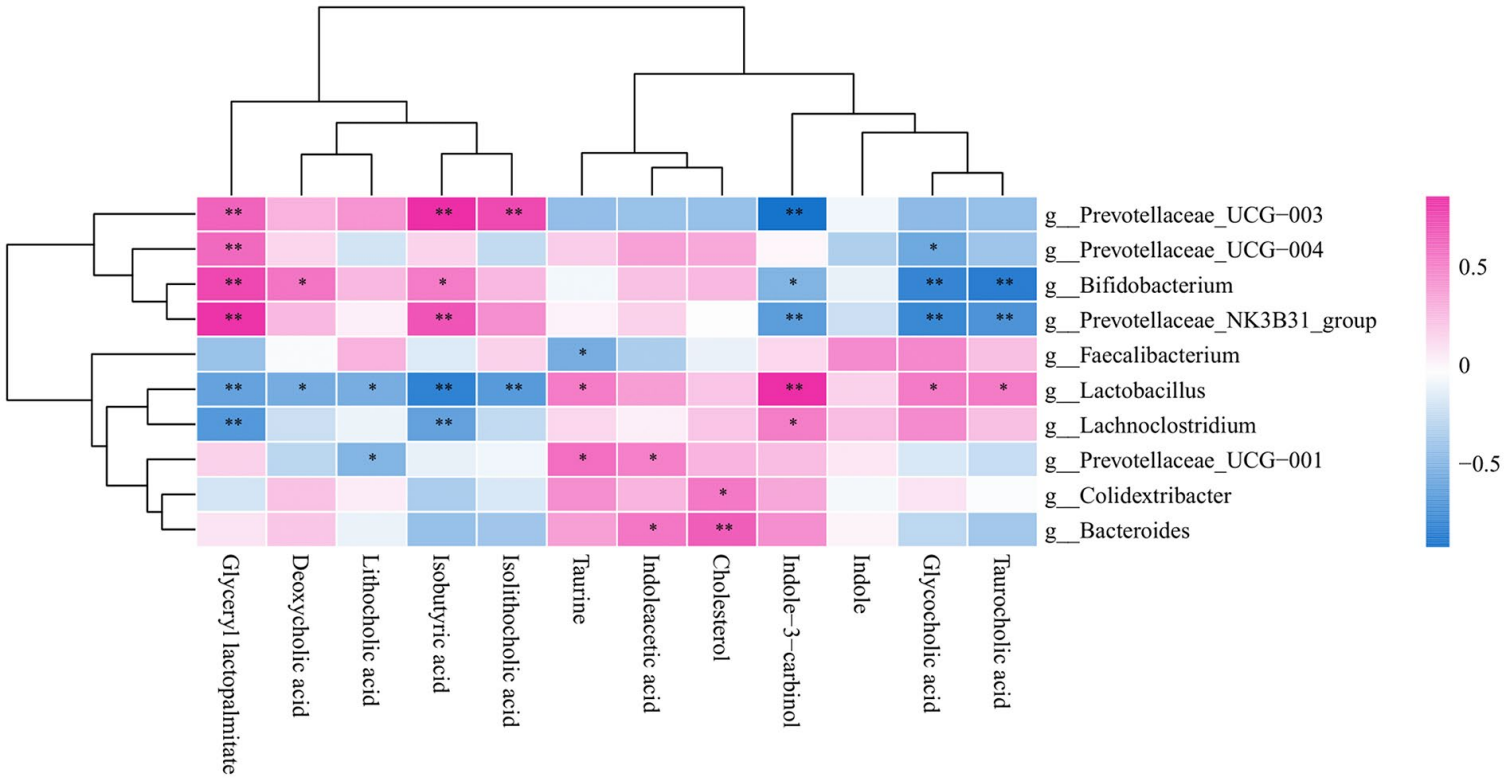
Correlation heat map of gut bacteria and metabolites.

Taurine and taurocholic acid can increase in the number of *Lactobacilluscae* and *Prevotellaceae_UGG-001*. The association between metabolites and gut microbiota showed that taurine was positively correlated with lactic acid bacteria, consistent with previous experimental results (Figure 7).

### Mechanism of taurine

**Figure 8:**
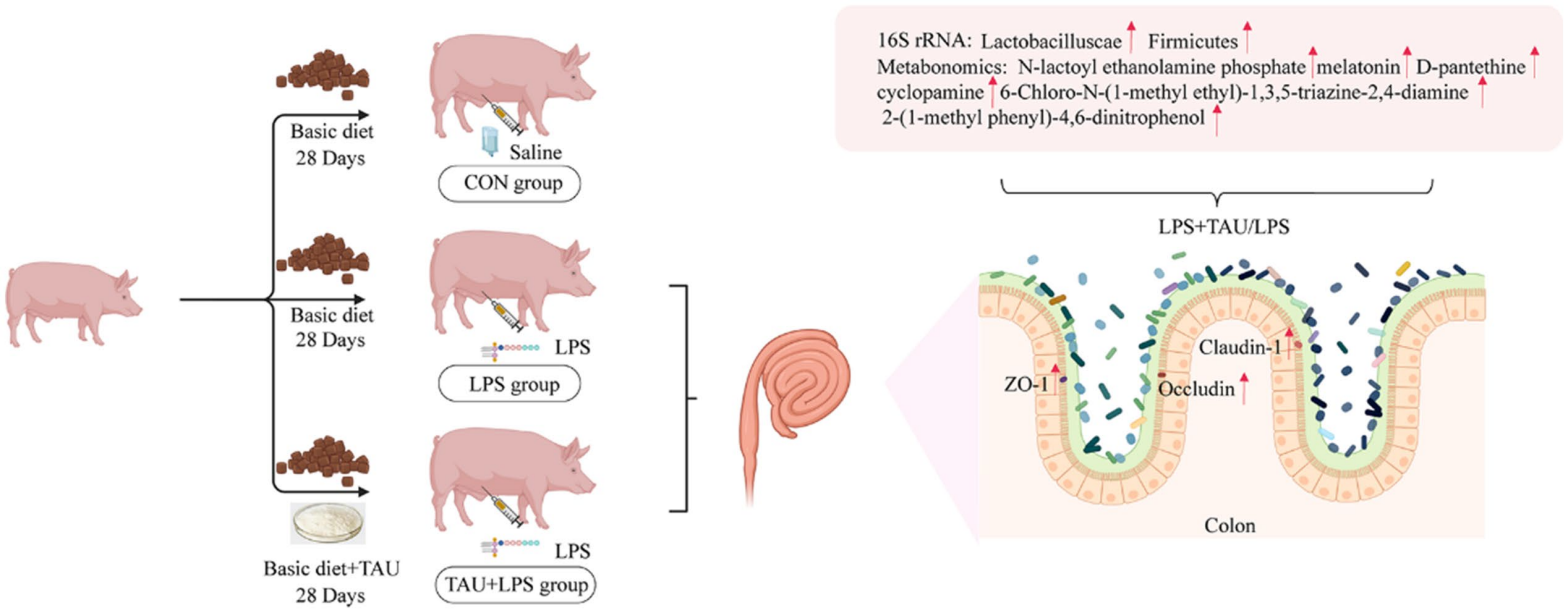
Mechanism of taurine.

Adding taurine to the base diet improves the colonic health and growth performance of piglets with diarrhea by improving the colonic tight junction mRNA and protein expression and regulating the content of microbial components and metabolites in the colon (Figure 8).

## Discussion

The intestinal barrier is the body’s first barricade to block the entrance of external toxins and can effectively block the entrance of antigens and harmful microorganisms. The tight junction comprises a multiprotein complex containing Occludin, Claudin-1, and ZO-1 (Pinton et al., 2010). Ensuring sustainable production in economic animals, gut health is a prerequisite since the gut plays an essential role in food digestion and nutrient absorption (Fu et al., 2023). When the tightly knit structure and function are affected by various physiological and pathological stimuli, the integrity of the intestine’s barrier is compromised, allowing microorganisms and toxins to migrate throughout the body via the mucosa. This phenomenon is called “intestinal leakage”(Ulluwishewa et al., 2011). *Campylobacter* in the jejunum has been reported to affect the integrity of the intestinal tract by disrupting the intestinal Occludin and enhancing the intracellular pathway of *Campylobacter* (Bhat et al., 2018). Therefore, some enteric pathogens use tight junctions to disrupt the intestinal tract, allowing pathogenic bacteria, feed contaminants, and pathogens to enter the digestive tract. Some enteric pathogens, such as *Escherichia coli*, *Salmonella typhimurium*, *Campylobacter congenii* and *C. jejuni*, *Helicobacter pylori*, *Clostridium perfringens* and *C. difficile* significantly damage the tight junctions of the intestinal tract (Ewaschuk et al., 2008). At the same time, the integrity of tight junctions also plays an essential role in controlling cell growth, differentiation, and signal transduction. Several aspects of the mechanism of impaired barrier function have been demonstrated, including deacidification of Occludin, decreased expression of tight junctions, and activation of Rho GTPase and myosin light chain kinase (MLCK) for non-muscle myosin stimulation (Miller and Wolin, 1996).

With the continuing discoveries in modern microbiology, the influence of the gut microbiota on intestinal injury is increasingly becoming a research hotspot. In this experiment, intraperitoneal administration of LPS to weanling piglets significantly reduced expression of Occludin, ZO-1, and Claudin-1 proteins and mRNAs. At the same time, scanning electron microscopy results showed that the TAU + LPS group effectively improved the folds and sinks of the colon and significantly suppressed colonic damage compared to the LPS group. These effects were as expected and confirmed that taurine, as a rational and effective feed additive, plays an essential role in mitigating injury of the intestine induced by LPS and ensuring the integrity of the intestinal barrier. LPS caused severe damage to the large intestine of weanling piglets, resulting in decreased expression of intestinal tight junction protein and mRNA of *ZO-1*, *Occludin*, and *Claudin-1* and impaired intestinal barrier. However, this situation was completely restored when taurine was added to the basal diet, indicating that taurine prevents intestinal injury induced by LPS.

The gut microbiota is thought to be an effective barrier to infection. However, little research has been done to date on the physiological processes involving the microbiota. The gastrointestinal tract in the mammal is home to dense, rich, and highly adaptive microbial communities. These communities maintain and ensure the integrity of the epithelium and provide an effective barrier against pathogens in the gut. The ability of a microbial community to prevent the invasion of foreign microorganisms and the spread of pathogens is described as colony formation resistance (Olsan et al., 2017). However, complex virulence mechanisms and metabolic adaptations have evolved from enteric pathogens to successfully compete with symbiotic microorganisms and access to the niches for infection in mammals (Pifer and Sperandio, 2014). To address this issue, some studies have pointed out that the effect of sulfonic acids on gut microbes is closely related to animal resistance and not detrimental to the organism, a future breakthrough based on microbial therapy (Collard et al., 2021).

It is well known that antibiotics reduce and eliminate harmful bacteria that compete for nutrients with host microorganisms in the gut and promote animal growth. However, while these substances eliminate pathogenic bacteria, they also inhibit normal microbial levels in the gut and reduce the balance of the lactobacillus microbiota (Gaudier et al., 2004). For example, *Klebsiella pneumoniae* (KPN), as a pathogenic bacterium, can achieve dense colony formation in the lumen of the intestine and aggressively cross the barrier of the intestine to invade extraintestinal sites and cause systemic disease (Hsu et al., 2015). In the KPN mouse oral model, only the usual small group of *Deltaprotobacteria*, including sulfate-reducing bacteria, expanded up to 100-fold in the microbial population of infected mice (Markowiak-Kopeć and Śliżewska, 2020). It should be noted that taurine alone cannot affect the innate or adaptive immune response of *Laminaprotobacteria* and only slightly alters the expression of genes in the isolated epithelial cells. However, administration of taurine to sterile GF mice does not increase KPN resistance, whereas transplantation of taurine-trained fecal microbiota into sterile GF mice promotes more resistance to KPN and underscores the importance of the taurine-trained microbiota and indicates that taurine metabolism plays an essential role in the mouse microbiota formation (Martin et al., 2016).

In this study, data analysis of the chao1 and Shannon index revealed a marked up-regulation in the abundance and intestinal microbiota diversity in piglets treated with LPS and a marked down-regulation in species richness and diversity in the TAU + LPS group compared with the LPS group. Thus, the mechanism of action of LPS and taurine may be tightly correlated with the alterations in the intestinal microbiota, providing important insights for further elucidating the detoxification mechanism of taurine. In this experiment, 16s rRNA sequencing technology was used to show that the relative abundance of microorganisms in the colon of piglets significantly changed after feeding a taurine-supplemented basal diet. For example, compared to the LPS group, the relative amount of *Spirochaetota*, *Bacteroidota*, and *Prevotellaceae* significantly down-regulated in the TAU + LPS group, while the relative abundance of probiotics *Lactobacilliaceae* and *Firmicutes* significantly up-regulated. These bacteria regulate the process of intestinal injury induced by LPS, and the changes in their abundance are closely related to taurine alleviating LPS-induced intestinal injury (Huynh and Zastrow, 2023).

Taurine can significantly ameliorate LPS-induced intestinal injury, which may be related to a decrease in *Bacteroides*, *Spirochaetota*, and *Prevotellaceae* in the colon and an up-regulation of the relative amount of *Firmicutes* and *Lactobacillaceae*, and taurine was demonstrated to play an essential role in alleviating LPS-induced intestinal damage. In addition, the relative amount of *Prevotellaceae* and *Streptococcaceae* was markedly up-regulated in the LPS group compared with the CON group, indicating that the relative amount of *Prevotellaceae* in the TAU + LPS group piglets was markedly decreased, alleviating LPS-induced damage to the colon, and *Prevotellaceae* pathogenicity of piglet colon could be proved. The increase of beneficial *Lactobacilli* plays a positive role in intestinal injury induced by LPS. Metabolomic analysis of colon contents showed that *Melatonin*, *6-Chloro-N-(1-methyl ethyl)-1,3,5-triazine-2,4-diamine*, 2*-(1-methyl propyl)-4* and *6-dinitrophenol* were markedly down-regulated and *Benazeprilat* was markedly up-regulated in the LPS group compared with the CON group. In the TAU + LPS group, the content of the above 5 metabolites was markedly altered compared with the LPS group. LPS-induced intestinal injury is correlated with a decrease in these metabolites, and taurine was found to reduce LPS-induced injury to the colon of weanling piglets by increasing the content of these metabolites. This study, in the analysis of microbiota and metabolite correlation, there were three main groups with increased numbers of beneficial bacteria, including *Faecalibacterium*, *Lactobacillus* and *Lachnoclostridium*. These beneficial bacteria produce active substances such as *taurine*, *indoleacetic*, *cholesterol*, *Glycocholic acid*, which form biological and chemical barriers in the digestive tract, preventing pathogenic bacteria from colonising and multiplying (Buffa et al., 2022).

The KEGG pathway map of differential metabolite enrichment showed that 14 differential metabolites were enriched on protein digestion and absorption (*p* = 0.01105). The *Slc1a1* and *Slc7a7* genes are located in this pathway, suggesting that taurine may improve intestinal function in piglets by regulating the expression of *Slc1a1* and *Slc7a7* genes (Jiao et al., 2015). In future studies, taurine can be added to promote the proliferation of intestinal epithelial cells in piglets to alleviate intestinal atrophy caused by weaning stress (Yin et al., 2014; Li et al., 2016) (Supplementary Figure S2).

## Conclusion

LPS disrupts intestinal microorganisms and metabolites and affects intestinal barrier function. Preventive addition of taurine improves the intestinal environment and mechanical barriers of weaned piglets by increasing the levels of *Lactobacillus porcineus, L. microgroups and L. acidophilus group, Clostridium perfringens and C. rumenii*, as well as the metabolites *Melatonin, 6-Chloro-N-(1-methyl ethyl)-1,3,5-triazine-2,4-diamine, 2-(1-methyl propyl)-4 and 6-dinitrophenol*. Thus, the results of the present study suggest that taurine can be used as a feed additive to counteract LPS-induced intestinal mechanical barrier damage in weaned piglets by increasing the number of beneficial microorganisms in the gut and influencing intestinal metabolites.

## Data availability statement

The datasets presented in this study can be found in online repositories. The names of the repository/repositories and accession number(s) can be found below: NCBI-PRJNA1036522.

## Ethics statement

The animal study was approved by the Animal Ethics Committee of Shenyang Agricultural University, Shenyang, China (No. 202006045). The study was conducted in accordance with the local legislation and institutional requirements.

## Author contributions

D-dZ: Writing – original draft. Y-dG: Writing – review & editing. CL: Writing – review & editing. Z-zF: Writing – review & editing. D-qY: Conceptualization, Writing – review & editing. MX: Data curation, Writing – review & editing. J-yD: Software, Writing – review & editing. X-xW: Writing – review & editing. Y-xL: Writing – review & editing. G-fW: Writing – review & editing. YF: Writing – review & editing. J-mH: Writing – review & editing. S-mL: Project administration, Writing – review & editing. J-cY: Project administration, Writing – review & editing.

## Supplementary material

SUPPLEMENTARY FIGURE S1 Microbial composition at the level of each sample intestinal segments.

SUPPLEMENTARY FIGURE S2 The KEGG pathway map of differential metabolite enrichment.
