# Supplementary figures and images for: Dietary taurine effect on intestinal barrier function, colonic microbiota and metabolites in weanling piglets induced by LPS
Source: Front Microbiol. 2023 Dec 22;14:1259133. doi: 10.3389/fmicb.2023.1259133 (PMC10770862; doi:10.3389/fmicb.2023.1259133)

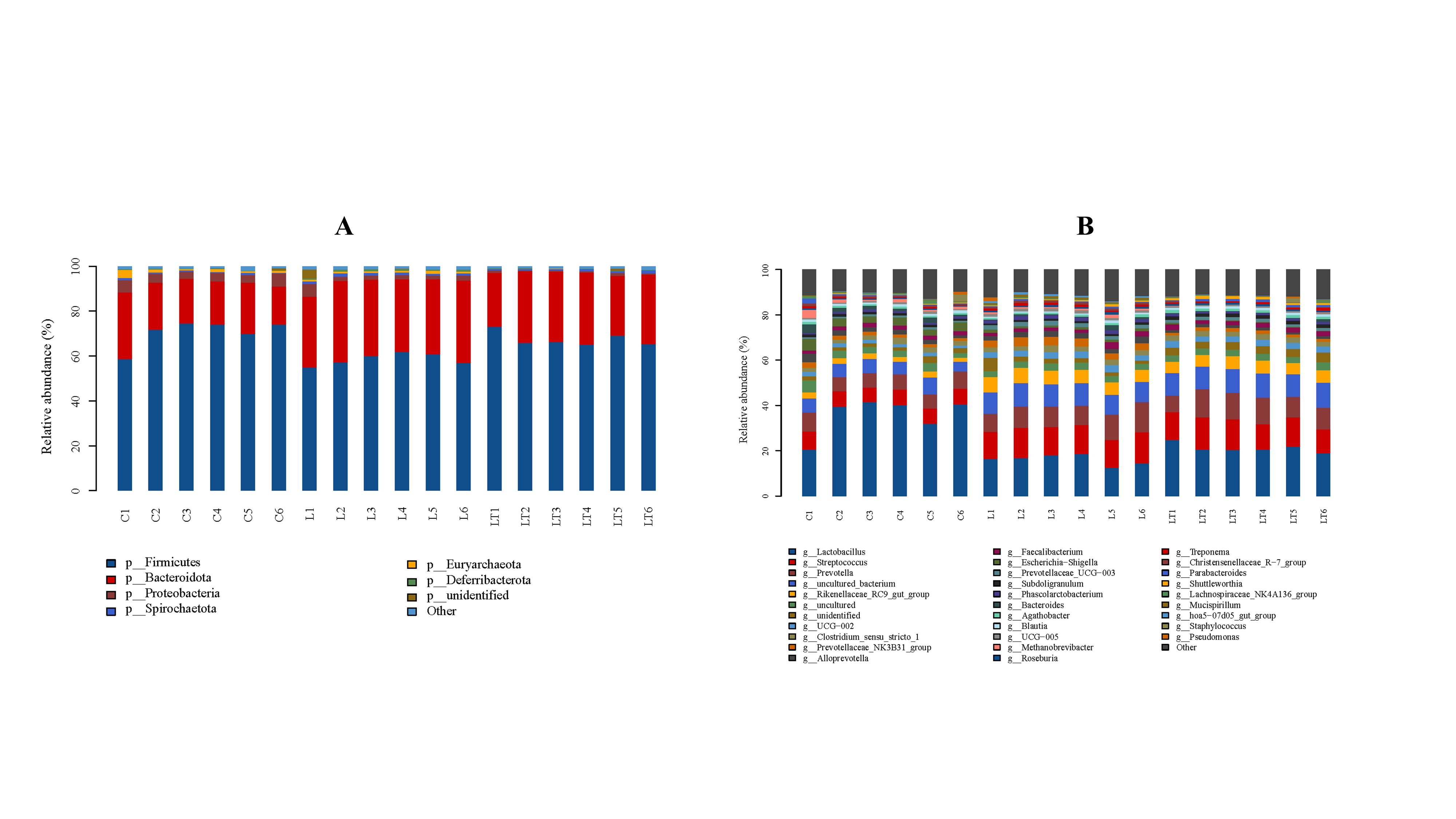

Supplement: SUPPLEMENTARY FIGURE S1 — Microbial composition at the level of each sample intestinal segments. [file Image_1.tif]

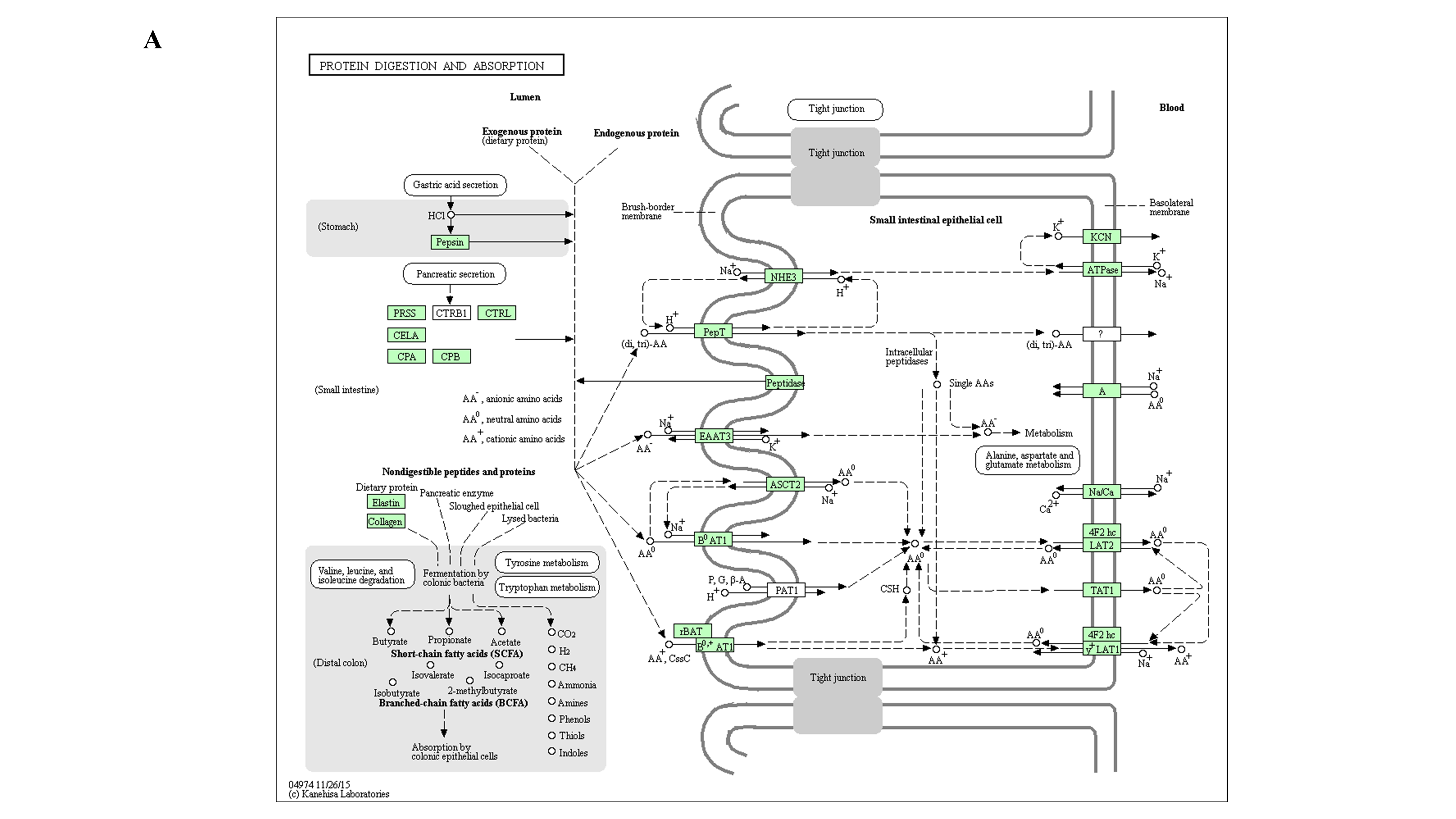

Supplement: SUPPLEMENTARY FIGURE S2 — The KEGG pathway map of differential metabolite enrichment. [file Image_2.tif]
