# Supplementary material for: Dietary taurine effect on intestinal barrier function, colonic microbiota and metabolites in weanling piglets induced by LPS
Source: Front Microbiol. 2023 Dec 22;14:1259133. doi: 10.3389/fmicb.2023.1259133 (PMC10770862; doi:10.3389/fmicb.2023.1259133)
Supplement: Supplementary file 3 [file Table_1.docx]

| Gene | Primer sequences（5’ to 3’） |
| --- | --- |
| *ZO-1* | F：GGAGGATGCTGTTGTCTCGG  R：GAGGATGGTCACACCGTGGT |
| *Claudin-1* | F：CCATCGTCAGCACCGCACTG  R: GACACGCAGGACATCCACAGC |
| *Occludin* | F：AAGGTTCCATAGCCTCGGTC  R：ATGCTTTCTCAGCCAGCGTA |

Supplementary Table. S1 The primers of target genes.
