# Supplementary material for: Dietary taurine effect on intestinal barrier function, colonic microbiota and metabolites in weanling piglets induced by LPS
Source: Front Microbiol. 2023 Dec 22;14:1259133. doi: 10.3389/fmicb.2023.1259133 (PMC10770862; doi:10.3389/fmicb.2023.1259133)
Supplement: Supplementary file 4 [file Table_2.docx]

Supplementary Table. S2 The percentage of the various groups that were identified as markers (%)

| Name | CON (mean±SD) | LPS (mean±SD) | LPS+TAU (mean±SD) |
| --- | --- | --- | --- |
| *Ruminococcaceae*  *wallaby_gut_metagenome*  *Lactobacillus_reuteri* | 3.97±0.002  0.08±0.001  4.89±0.011 | 4.29±0.007  0.08±0.001  3.21±0.003 | 5.14±0.005  0.34±0.001  6.63±0.005 |
| *Clostridium_sensu_stricto_6* | 0.13±0.001 | 0.08±0.001 | 0.47±0.001 |
| *Eubacterium_coprostanoligenes_group* | 2.07±0.003 | 1.75±0.003 | 2.86±0.003 |
