# Supplementary material for: Dietary taurine effect on intestinal barrier function, colonic microbiota and metabolites in weanling piglets induced by LPS
Source: Front Microbiol. 2023 Dec 22;14:1259133. doi: 10.3389/fmicb.2023.1259133 (PMC10770862; doi:10.3389/fmicb.2023.1259133)
Supplement: Supplementary file 5 [file Table_3.docx]

Supplementary Table. S3 Significant endogenous metabolites LPS VS LPS+TAU

| Name | Mean LPS | Mean LPS+TAU | P_Value | q_Value |
| --- | --- | --- | --- | --- |
| N-Lactoyl ethanolamine phosphate | 1.6144285e-05 | 0.01272 | 1.1427e-09 | 2.6708e-08 |
| Melatonin | 0.00046 | 0.17248 | 8.8729e-15 | 5.8915e-12 |
| 2-(1-Methylpropyl)-4,6-dinitrophenol" | 0.00017 | 0.02215 | 1.4582e-06 | 8.8498e-06 |
| 6-Chloro-N-(1-methylethyl)  -1,3,5-triazine-2, 4-diamine | 0.01397 | 0.52750 | 4.1582e-05 | 0.00015685 |
